# Investigating the investigations: senior healthcare leaders’ perspectives on the organisational value, burden and effectiveness of serious adverse event investigations

**DOI:** 10.1108/JHOM-12-2025-0895

**Published:** 2026-03-03

**Authors:** Jarrid Brunton, Sharon Lawn

**Affiliations:** College of Medicine and Public Health, Flinders University, Adelaide, Australia; Flinders University, Adelaide, Australia

**Keywords:** Patient safety, Serious adverse events, Investigations, Healthcare leadership, Governance, Organisational learning, Root cause analysis (RCA), Accountability, Just culture, Health services management

## Abstract

**Purpose:**

Serious adverse event (SAE) investigations are a routine feature of healthcare quality assurance, although concerns persist regarding their effectiveness, known costs and contribution to organisational learning. This study explores senior healthcare leaders’ perspectives on SAE investigation practices within Australian public healthcare organisations, with a focus on organisational value, resource implications, costs incurred to conduct and the impact of legislated protection.

**Design/methodology/approach:**

Semi-structured interviews were conducted with 15 senior healthcare leaders across four Australian jurisdictions. Data were analysed using reflexive thematic analysis.

**Findings:**

Leaders reflected on SAE investigations methods as complex processes that balance investigation, quality assurance and accountability, yet deliver mixed outcomes in terms of prevention, often characterised by repetitive recommendations and limited dissemination of learning. Despite their perceived value in providing organisational assurance, investigations were described as costly due to heavy reliance on senior medical staff, with legislated protection facilitating openness while simultaneously limiting transparency and sharing of organisational learning.

**Research limitations/implications:**

Reporting leaders’ perceptions of how these system pressures and governance arrangements shape investigation processes and the use of findings, this study contributes new knowledge to debates on safety governance and organisational learning in complex healthcare organisations.

**Practical implications:**

Healthcare leaders could consider more flexible, proportionate investigation methods, invest in investigator capability, clarify guidance on legislated protection and systematically monitor investigation costs and outcomes to support sustainable commissioning of these investigations.

**Originality/value:**

This study offers original qualitative insight into senior healthcare leaders’ experiences of SAE investigations within Australian public health systems. Through in-depth interviews, it extends a literature dominated by quantitative and method-focused studies by examining how investigations are understood, governed, and enacted in practice. The findings illuminate the leadership work involved in balancing accountability, organisational learning, ethical obligations, and resource constraints. By revealing how system pressures and governance arrangements shape investigation processes and the use of findings, this study contributes new knowledge to debates on safety governance and organisational learning in complex healthcare organisations.

## Introduction

Healthcare organisations worldwide continue to experience high rates of adverse events, with serious adverse events (SAEs) representing the most severe and preventable forms of patient harm. International evidence consistently demonstrates that one in 20 patients are harmed during healthcare, with approximately half of this harm considered preventable (Panagioti *et al*., 2019; WHO, 2021). In Australia, National data indicates that hundreds of thousands of hospital admissions each year involve an adverse event, with sentinel events, such as inpatient suicide and wrong-site/patient surgery, continuing to occur despite stringent regulatory and governance oversight (AIHW, 2025; ACSQHC, 2022).

In response to serious patient harm, healthcare organisations are typically required to undertake formal investigations, with Root Cause Analysis (RCA) the main and most widely used approach internationally and within Australian public health systems (ACSQHC, 2017). RCA originated in high-risk industries such as aviation, nuclear power and manufacturing, where it was developed as a method for analysing system failures and preventing recurrence through structured investigation (Kellogg *et al*., 2017). This structured, systems-oriented investigation method is intended to move beyond individual error and identify underlying contributory factors that may have enabled an incident to occur. Although specific models vary (Hibbert *et al*., 2018), RCA processes generally involve several core stages: initial event notification, assembly of an investigation team; data collection through document review, interviews and timeline reconstruction; identification and analysis of contributory factors across individual, task, team, organisational and system levels; development of recommendations and preparation of a formal report (Peters and Eng, 2021; Park *et al*., 2023; Wood *et al*., 2024).

In practice, RCA is positioned as a learning-oriented governance tool that seeks to shift analysis away from individual error and toward the identification of systemic contributors to harm, with the intention of informing organisational improvement. RCA has been subject to longstanding critique within the patient safety literature, with ongoing research questioning whether RCAs reliably achieve these aims. Critiques highlight variability in methodological quality, limited use of systems thinking, over-reliance on linear causation (Braithwaite *et al*., 2017), and the frequent generation of weak recommendations (Hibbert *et al*., 2018) that focus on superficial policy changes rather than fundamental structural reform (Peerally *et al*., 2017; Martin-Delgado *et al*., 2020; Lea *et al*., 2023).Concerns have also been raised regarding the substantial time and resource burden associated with RCA processes, alongside limited evidence that they reduce recurrence of SAE in highly complex systems, such as healthcare (Haylor *et al*., 2024).

These critiques are increasingly situated within broader debates in safety science regarding Safety-I and Safety-II paradigms (Hollnagel, 2018). Traditional RCA methodological approaches are closely aligned with Safety-I thinking, which conceptualises safety as the absence of errors and seeks to understand why things go wrong through retrospective analysis of failure. Within this paradigm, investigations focus on identifying causes, deviations and corrective actions following harm. In contrast, Safety-II reframes safety as the capacity of systems to succeed under varying conditions, emphasising how everyday work is adapted, why things usually go right, and how system resilience can be strengthened. Proponents argue that Safety-II offers a more realistic and generative framework for understanding complex healthcare systems and for supporting learning that extends beyond isolated events (Dekker and Breakey, 2016).

Calls to transition toward Safety-II approaches have highlighted the need to reconsider the heavy reliance on SAE investigations (Hollnagel *et al*., 2015; Braithwaite *et al*., 2017). Such a transition has significant implications for leadership, as it requires senior leaders to rethink how safety is conceptualised, operationalised and how investigative resources are aligned to meet this transition (Spanos *et al*., 2024). Understanding senior healthcare leaders’ perspectives is therefore critical at this juncture. Leaders play a central role in shaping whether, and how, organisations move beyond Safety-I–dominated investigation practices toward more balanced approaches that incorporate Safety-II principles.

Beyond safety science theory, SAE investigations are embedded within organisational and legal contexts that influence information sharing and disclosure. Legislative protection frameworks, designed to limit disclosure of SAE investigation material, are intended to promote staff candour and psychological safety but have increasingly been recognised as legal mechanisms that restrict transparency and constrain information sharing and open disclosure (Dekker and Breakey, 2016; O’Hara *et al*., 2018). Within this legal context, leaders must navigate competing obligations to support staff participation, meet regulatory requirements and respond to the information needs of families, boards and the wider system (Seys *et al*., 2023).

Leaders’ views on SAE investigations provide insight into the readiness, challenges and governance dilemmas shaping this transition. Senior healthcare leaders occupy a critical position within this landscape (Armutlu *et al*., 2020), as they are responsible for commissioning investigations, allocating resources, overseeing governance responses and assuring boards, regulators and the public that appropriate action has been taken (Spanos *et al*., 2024). Despite their pivotal roles, there is limited empirical research examining how leaders themselves perceive the organisational value, challenges, burden and effectiveness of SAE investigations. Understanding these perspectives is essential to informing more proportionate, learning-oriented and sustainable investigation systems. This study contributes fresh insights from a qualitative study of the perceptions of 15 senior healthcare leaders of Australian public healthcare organisations. It shows their work managing the trade-offs between the demands of accountability, organisational learning, ethical obligations, and resource constraints.

## Methods

### Study design and methodological orientation

This study was guided by a pragmatic epistemological stance and an interpretivist theoretical lens, informed by Crotty’s (1998) research design framework. Crotty’s framework provided a coherent structure to ensure alignment between the study’s philosophical assumptions, methodological approach, and analytic processes. A qualitative interpretive approach was adopted to capture how leaders understand, experience and make sense of real world investigation practices within complex organisational and governance environments, recognising that SAE investigations are socially, organisationally and politically situated practices shaped by regulatory requirements, professional norms and leadership responsibilities. This approach enabled exploration not only of what investigation practices occur, but how leaders interpret their value, burden and effectiveness. The study was informed by constructivist assumptions, recognising that participants’ accounts represent situated interpretations of organisational reality rather than objective truths. This orientation was appropriate given the study’s focus on leadership sensemaking, governance decision-making and the influence of organisational context on SAE investigation practices.

### Setting

This study was conducted within Australian public healthcare organisations operating under Local Health Network (LHN) governance structures (ACSQHC, 2017). The term “Local Health Network” is used generically in this study to describe state- and territory-based public health service organisations across Australia, also referred to in some jurisdictions as regional services, hospital networks or health districts. LHNs are publicly-funded statutory entities responsible for the planning, governance and delivery of hospital and health services within defined geographic regions. These organisations provide a range of acute and specialist services and operate within comparable regulatory, governance and accountability frameworks. They are accountable to state and territory health departments for clinical governance, quality and safety, including the oversight of SAE investigations. Eastern states were deliberately excluded, as similar research examining SAE investigation practices was being conducted concurrently in those jurisdictions; this decision was made to minimise duplication and reduce potential confusion for organisations and participants.

### Study participants

Participants were eligible for inclusion if they were working at senior executive or high-level governance positions within their organisation and held responsibility for the oversight of clinical governance and patient safety. This included executive directors, directors responsible for clinical governance and quality, senior clinical governance leads, and senior managers with responsibility for clinical commissioning, safety oversight or system-level assurance. Participants were required to have current responsibility for commissioning, overseeing or approving SAE investigations at an organisational or jurisdictional level.

### Sampling and recruitment

Purposive sampling was used to recruit senior healthcare leaders. Initial participants were identified through their involvement in previous health services research conducted by the lead researcher; however, responses to the request to participate in the further study were limited. A snowball sampling strategy was therefore employed, whereby participants recommended other leaders with relevant expertise and responsibilities. This approach enabled access to senior leaders with deep organisational knowledge across multiple LHNs and jurisdictions. Recruitment continued until thematic saturation was achieved, defined as the point at which no substantively new concepts or perspectives emerged. Sample size was restricted by ethics approval requirements, as several LNHs required individual site-specific approvals – a lengthy process that would have taken many months to achieve across LHNs. In total, fifteen senior healthcare leaders participated in the study, drawn from 116 LHNs.

### Study instrument development

The interview guide was deliberately designed to provoke open conversation, encouraging participants to reflect freely and shape the direction of discussion rather than respond to narrowly defined questions. This approach allowed flexibility during interviews, enabling probing, clarification and follow-up questions to be tailored to individual participants and organisational contexts, while maintaining alignment with the core areas of inquiry. The open-ended design supported exploration of participants’ meanings, interpretations and decision-making processes, rather than constraining responses to predefined categories.

The interview guide was reviewed iteratively during early data collection to ensure questions were comprehensible, relevant and capable of eliciting detailed responses, without substantive changes to the focus or intent of the study. Questions were designed to elicit leaders’ experiences, perceptions and reflections on the value, burden and effectiveness of SAE investigations. Interview questions are presented in Table 1.

**Table 1:** Semi-structured interview guide questions

Research domain	Interview question
Legislated protection	What are your thoughts on legislated protection, to prevent the disclosure of information following a serious adverse event incident/investigation?
Investigation costs	What are your views on the organisational costs/hrs associated with conducting serious adverse event investigations?
Perceptions	What do you see as the key benefits of conducting serious adverse event investigations? What do you consider to be the main weaknesses or limitations of current serious adverse event investigation processes? What changes or improvements are needed to enhance serious adverse event investigations? What are your thoughts regarding the notion of blame when completing serious adverse event investigations? What are your thoughts on sharing recommendations to support organisational learning? What are your thoughts on investigations and the impact on teamwork?

### Data collection

Data were collected through semi-structured, one-to-one interviews with senior healthcare leaders. Interviews were conducted over a 6 month period (April and October 2024) via Microsoft Teams, reflecting participants’ senior leadership roles, high workload and geographic dispersion across Australia, and enabling participation across multiple jurisdictions with minimal disruption to operational responsibilities. All interviews were conducted by the lead researcher to ensure consistency in approach and questioning. The lead researcher has professional expertise in healthcare quality, safety and governance, supporting informed engagement with participants while maintaining reflexive awareness of potential power dynamics. The researcher held no managerial authority over participants and had no supervisory or reporting relationship with them. An interview guide was developed by the research team based on prior literature examining SAE investigations, healthcare governance, leadership and organisational learning. The interview guide consisted of core topic areas focused on investigation methods and processes, perceptions of effectiveness and learning, resource and time burden, governance oversight and the role of legislated protection. Interview questions were intentionally open-ended to allow flexibility during interviews and to support probing, clarification and exploration of issues raised by participants. Interviews were deliberately time-limited to approximately 30 min, following agreement with and assurance to participants that interviews would not exceed this duration or compromise their competing workplace leadership demands. As a result, not all participants were asked every interview question; however, core topic areas were consistently explored across interviews to ensure comparability of data. Subject to informed consent, all interviews were audio-recorded and transcribed verbatim.

### Data analysis

Analysis was guided by the six-phase framework outlined by Naeem *et al*. (2023), which is largely based on the reflexive thematic analysis approach described by Braun and Clarke (2006). Coding was supported by NVivo software to facilitate systematic data management. First, all transcribed interviews were checked for accuracy against the recording. The lead researcher then read and re-read the transcripts to achieve familiarisation with the data, noting initial ideas and reflections. Second, initial codes were generated inductively across the dataset, focusing on semantic features of the data relevant to leaders’ experiences of investigation practices. In the third phase, codes were examined for patterns and relationships and collated into candidate themes. This involved iterative movement between coded extracts and the full dataset to ensure that themes captured shared meanings while remaining grounded in participants’ accounts. In the fourth phase, candidate themes were reviewed and refined through ongoing dialogue within the research team and a SAE subject matter expert with the SA Government Department for Health and Wellbeing. Themes were assessed by the team for internal coherence and external distinction, ensuring meaningful differentiation between themes. In the fifth phase, themes were clearly defined and named through analytic writing, clarifying their core organising concepts and relevance to the research question. Finally, the analysis was synthesised into a coherent narrative, supported by illustrative quotations, and reported by the lead researcher and two other researchers of the team with expertise in quality improvement, leadership and qualitative research methods.

### Ethical approval

Ethics approval was obtained from the South Australian Department for Health and Wellbeing Human Research Ethics Committee (HREC reference: 2023/HRE00293) in December 2023. All required site-specific approvals were obtained, and no ethical issues, protocol deviations, or approval constraints were encountered during the conduct of the study. All participants provided informed consent prior to interview.

### Rigour and trustworthiness

Rigour and trustworthiness were addressed through credibility, transferability, dependability and confirmability (Houghton *et al*., 2013). Credibility was enhanced through systematic development of the interview guide informed by relevant literature, sustained engagement with the data, and iterative analytic dialogue within the research team. Themes were refined through regular supervision meetings, supporting reflexivity and interpretive depth. External scrutiny was provided by presenting themes to an independent senior healthcare leader within the South Australian Department of Health to assess coherence, plausibility and practical relevance. Transferability was supported through detailed description of the study context, organisational settings and participant characteristics. Dependability was strengthened through transparent reporting of data collection and analysis procedures, including use of a recognised reflexive thematic analysis framework and documentation of analytic decisions. Confirmability was addressed by grounding interpretations in participants’ accounts, illustrating themes with verbatim quotations in italics and participant identifiers, and through ongoing reflexive discussion within the research team.

## Results

Fifteen senior healthcare leaders participated in the study. Participants included both female (*n* = 12) and male (*n* = 3) leaders and held executive and senior governance roles, including executive directors (*n* = 5), directors (*n* = 9) of clinical governance. Participants were drawn from public healthcare organisations across four Australian jurisdictions: South Australia (*n* = 8), Western Australia (*n* = 4), the Northern Territory (*n* = 1) and Tasmania (*n* = 1). Leaders represented metropolitan (*n* = 8), regional/rural (*n* = 3) and specialised health network (*n* = 2). Participant demographic characteristics are summarised in Table 2.

**Table 2:** Participant demographic characteristics

Variable	Categories	Participant responding ^*(n =15)*^
State	SA	9
	TAS	1
	WA	4
	NT	1
Position	Executive Director	5
	Director	8
	Manager	2
Professional background	Nursing	10
	Physician	3
	Corporate	2
Mode	Face to face	–
	MS Teams	14
	Telephone	1
Service type	Metropolitan Tertiary Health Network	8
	Specialised Health Network	5
	Rural and Remote Health Network	2
Gender	Female	12
	Male	3

Data analysis generated five interrelated themes that capture senior healthcare leaders’ experiences of SAE investigations. These themes were: (1) the hidden burden of resources; (2) questioning the impact of investigations; (3) assuring quality through investigation; (4) communication challenges under legislative constraints; and (5) the administrative cost of safety investigations. Together, these themes reflect the tensions leaders navigate between organisational assurance, learning and sustainability within contemporary investigation systems. A summary of the themes and their core organising concepts are presented in Table 3.

**Table 3:** Summary of themes and core organising concepts

Themes	Sub-themes	Theme expansion
1. The hidden burden of resources	1. Re-routing limited resources	1. The Investigation process is labour-intensive and requires a group of staff to be redirected from clinical duties or responsibilities to investigate within a tight timeframe
	2. Uneven distribution of workload	2. The duties of investigating are not evenly distributed amongst investigators resulting in a disproportionate workload allocation
	3. Restrictive timeframes	3. Unrealistic completion deadlines hinder the ability to undertake a comprehensive investigation and lead to a hasty process that fails to respect the incident's victim(s)
2. Questioning the impact of investigations	1. Fails to prevent reoccurrence	1. Investigations and the associated recommendations fail to prevent the same incident type from reoccurring within the organisation
	2. Neglects contextual factors	2. Investigation reports fail to recognise organisational dynamic factors that contributed to the incident resulting in a lack of context within the findings and recommendations that failed to address the broader issues within the system
	3. Inadequately monitoring of outcomes/learnings	3. Recommendations are not adequately monitored and audited to measure effectiveness in preventing reoccurrence
	4. Ineffectiveness of sharing learnings	4. Investigations adopt a single incident perspective and report similar incidents in silos. This method fails to incorporate a thematic approach or analysis of incident clusters
	5. Focus on a single incident perspective	5. Learnings and recommendations are specific to the site the incident occurred. Consequently, sharing recommendations is poorly received and often considered out of context to another health service
	6. Varying quality of reports/outcomes	6. Final reports vary in quality and recommendations. This variation is often based on the skill and experience of the individual team members
	7. Generates blame	7. Investigations primarily focus on exploring “error”, which drives a sense of persecution, failure, and blame within the workforce. The sense of blame contributes to underreporting of similar incidents and fuels disconnection from the workforce
3. Assuring clinical quality through formal investigation and review mechanisms	1. Evidence of remedial action	1. Investigations provide a sense of assurance to the community, victim, and workforce that the organisation is addressing the incident seriously and taking remedial action to prevent reoccurrence
	2. Independent scrutiny/exploration	2. A comprehensive investigation provides a level of independence and scrutiny, allowing the exploration of organisational and system issues with the aim of achieving organisational-wide quality improvement
	3. Identification of quality improvement opportunities	3. Investigations provide a systematic and structured approach, allowing investigators to work through the chain of events that led to an incident and identify a root cause, which can then be used to develop organisational learning and quality improvements
4. Communication challenges under legislative constraints	1. Restricted communication	1. Restricted communication leads to a perception that the organisation is attempting to conceal certain aspects of the incident, hindering the organisation’s ability to provide meaningful open disclosure to the patient, family, and clinical team involved in the incident
	2. Increases confidentiality	2. Legislated privilege creates a sense of security and allows staff to speak openly without fear of repercussions or being personally identified. Hence, supporting a deeper exploration of the circumstances of the incident and the identification of the underlying cause(s)
5. The administrative cost of safety investigations	1. High cost of professional groups	1. Highly paid professionals are predominantly involved in overseeing investigations and contribute towards the costs incurred to investigate and present the findings
	2. Rigorous reporting requirements	2. Investigations are subject to significant reporting requirements across multiple forums within an organisation, such as governance committees and board-level enquiries. These forums are highly represented by senior clinical staff and high-paid professional groups

### Theme 1: the hidden burden of resources

Leaders described the SAE investigation process as highly labour-intensive, requiring multiple staff to be redirected from clinical duties or existing responsibilities to complete investigations within mandated and often tight timeframes. This workload was not perceived to be evenly distributed, with investigation responsibilities frequently falling to a small group of experienced investigators, resulting in a disproportionate allocation of effort and cumulative burden.

Resourcing is a huge thing ….We don't have enough resourcing to meet the level. Everyone's working 100% over capacity. (P1)

It’s just added on top of everything else — there’s no extra time or capacity. (P7)

Every serious incident investigation pulls people away from patient care and leadership work for weeks, and that cost is never visible at board level. (P4)

Constraints on the people that we're asking to contribute are so significant. I spent two weeks just trying to get a panel (investigation team) together. (P4)

It's all done through existing staff. We don't engage or contract out any of the investigation process. So basically, they (staff) will be facilitators of the RCA process itself. (P2)

Participants highlighted that mandated investigation timeframes were often unrealistic and prioritised procedural completion over reflective learning. Compressed timelines were seen to constrain opportunities for meaningful engagement with staff and families and to limit the depth of analysis, encouraging rushed processes that risked overlooking contextual factors. As a result, investigations were sometimes perceived as insufficiently respectful of those affected by the incident, including victims and their families.

We want to encourage that culture of patient safety but facilitating and the administrative work behind it, it's pretty near impossible to meet the timelines and KPIs that are set out for us. (P1)

We have 28 days to do these investigations and complete them by which is the shortest time across Australia. (P8)

We’re racing deadlines rather than learning properly from the event. (P1)

### Theme 2: questioning the impact of investigations

Leaders expressed growing scepticism about whether SAE investigations consistently achieve their intended purpose of preventing recurrence and improving patient safety, and questioned whether the substantial organisational effort invested translates into meaningful and sustained change. Investigations were frequently described as producing familiar narratives and recurring recommendations, contributing to perceptions that they operate primarily as compliance activities rather than as mechanisms for deep system learning.

In six months’ time, the same problem has occurred, and it's like, well, that didn't do anything, did it? (P3)

We've got multiple RCAs across our organisation, and they all are looking at acute deterioration of some description. (P14)

We get very focused on the, the investigation and making the recommendations and don't spend quite as much energy the other at the next step. (P14)

Leaders emphasised that while recommendations were commonly endorsed, there was limited organisational capability to share, implementation or evaluate.

I have gone through all the recommendations, and I don't even know what half of them mean. (P13)

We've got to get better at following up the recommendations and closing the loop. (P9)

No one checks later whether the recommendations actually made a difference. (P12)

I don't think they are meaningfully shared. We do have some mechanisms for sharing them. I don't think that they get down to the to the people on the floor; probably they're the ones that probably need to see or hear about the recommendations. (P2)

Leaders attributed the limited impact of SAE investigations to variability in investigator capability, particularly in systems thinking and recommendation development, noting that final report quality and the strength of recommendations often depended on the skill and experience of individual team members.

The outcome depends entirely on who’s leading the investigation. (P5)

Quality of reviews is so variable; they are really hit and miss. (P9)

### Theme 3: assuring clinical quality through formal investigation and review mechanisms

Despite concerns about burden and effectiveness, leaders consistently articulated a strong assurance function associated with SAE investigations. For senior leaders, the assurance value of investigations extended beyond learning to include maintaining trust with boards, regulators, families and the broader public. Investigations were therefore understood as critical governance instruments, even when their contribution to improvement was uncertain.

Families or … loved ones, other professionals … have an expectation that there will be an external review of this incident. (P9)

Assuring the organisation that we're taking it seriously and that we have mobilised our response. (P2)

It provides us with a level of protection because we’ve done something. (P2)

Doing a deeper dive into serious investigations provides you with system issues … to address. (P15)

Independent scrutiny, by external or independent investigations, were viewed as particularly valuable in reinforcing legitimacy, especially in high-profile or contested cases.

We get staff members from other health services …. as experts around the table, which adds rigor and independence to the investigation. (P10)

Including people from overseas if we need to bring on to the panel so that we've got some independence in our review. (P12)

### Theme 4: communication challenges under legislative constraints

Leaders described communicative and ethical tensions when SAE investigations were conducted under legislated protection. While confidentiality frameworks were seen to promote staff candour and deeper exploration of incident circumstances, restricted communication was also perceived as limiting transparency and, at times, creating an impression of organisational concealment. These constraints were viewed as hindering meaningful open disclosure, restricting the sharing of contextual learning, and complicating relationships with patients, families, and clinical teams, particularly recognising the healthcare industry focus on transparency.

The recommendations were the only thing public and often the recommendations didn't have the context because all you were allowed was the newspaper style. (P3)

Protected under the Act …, just makes it really difficult because then how do we share lessons? (P8)

Removal of protection …..It allows people to see real cases, hear about real recommendations and know what's happening rather than having a secret squirrel society. (P3)

Participants described an ongoing tension between encouraging open staff participation and safeguarding confidential disclosure, balanced with open reporting that would enable broader organisational learning.

It helps staff speak openly, but it limits how much learning we can share. (P11)

Staff feel safe so they are able to speak more freely without risk of consequence in a protected environment. (P12)

If we have screwed up … I think that the patients and their family have rights to access information around what went wrong, what we have done in response and how we've tried to remediate. (P5)

Leaders described challenges in balancing families’ expectations for openness with the legal constraints governing SAE investigations, noting impacts on both family relationships and the wellbeing of the workforce.

Families want answers, but legally there are limits. (P6)

Removal of privilege would be a good opportunity to raise that transparency and share those improvements more widely. (P1)

Privilege is just going to put up more walls than actually protect anybody. (P10)

### Theme 5: the administrative cost of safety investigations

Leaders raised concerns that SAE investigations have become increasingly administratively dense, characterised by multiple layers of reporting, review, and oversight. Participants described a progressive escalation of governance requirements, often driven by risk aversion and external scrutiny. Investigations were subject to extensive reporting across multiple organisational forums, including governance committees and board-level processes, which were heavily attended by senior clinicians and highly paid professional groups and consultants.

Layers of sign off … we get a lot of Executive team members to sign off on the final report. That means they've got to read it. (P3)

90 minutes, of a bunch of probably 12 people, of which five are psychiatrists. P11 The committee quorum is about 8 or 9, which means we've probably got about 17 people to draw from. (P9)

Leaders noted that the involvement of these roles contributed substantially to the overall cost of investigations. While such processes were intended to strengthen accountability, leaders questioned whether they meaningfully enhanced organisational learning or primarily functioned to manage organisational and reputational risk.

Consultants on $500.00 an hour, so you've got them for a number of hours (P12). Another added, A consultant psychiatrist … they're paid a lot of money. So, $13,000 isn't even going to cover them. (P1)

You don’t tend to have junior staff around the table … each meeting would be at least an hour and a half or longer; we would book at least two hours. (P9)

## Discussion

This study provides the first known qualitative examination of senior healthcare leaders’ perspectives on SAE investigations within Australian public healthcare networks. Through interviews with 15 senior leaders across four jurisdictions, the study illuminates how SAE investigations operate at the intersection of public accountability, organisational learning, resource constraint and legislative complexity. The findings reveal a dynamic and often contradictory set of expectations, whereby SAE investigations simultaneously function as mechanisms of accountability, learning and risk management, while also viewed as imposing a substantial operational burden (Hussain *et al*., 2019; Ramsey *et al*., 2022).

Participants consistently described SAE investigations as playing a critical role in preserving public confidence following serious harm, particularly in contexts characterised by uncertainty, heightened media scrutiny or perceived organisational defensiveness (Hall and Wolf, 2021; Hong and Oh, 2020; Souvatzi *et al*., 2024). In this context, investigations were viewed as visible demonstrations that adverse events are formally examined and acted upon, aligning with literature emphasising transparency and open disclosure as central to sustaining patient provider trust (Finkelstein *et al*., 2024; Manser and Staender, 2005; Prentice *et al*., 2020; Wood *et al*., 2024). At the same time, leaders emphasised that investigations serve important organisational risk-management functions, including mitigating reputational damage, managing media scrutiny and reducing legal exposure (Guerra, 2024; Hallam, 2002; Haylor *et al.*, 2024; Schiller and Prpich, 2014). This dual role reinforces evidence that healthcare leaders must continually balance patient-centred values with organisational risk imperatives, a tension increasingly highlighted within contemporary leadership literature (Al-Hussami *et al*., 2018; Mathew *et al*., 2024; Spanos *et al*., 2024; Viscelli *et al*., 2017).

Across interviews, the resource burden associated with SAE investigations emerged as the most persistent and multifactorial concern. Leaders described investigations as time-intensive, labour-intensive and emotionally demanding processes (Abdi and Ravaghi, 2017; Wallace *et al*., 2009). These accounts align with evidence that investigations often divert clinicians from frontline care (McHugh *et al*., 2024; Willis *et al*., 2023), impose unrealistic completion deadlines (Kwok and Mah, 2023), and require sustained involvement from senior clinicians whose high labour costs significantly increase organisational expenditure (Alexander *et al*., 2007; Pereira *et al*., 2025). Mandated investigation completion timeframes, or imposed key performance indicators (KPIs) were widely perceived as compromising analytic depth and validity while intensifying the above workforce pressures.

Resource burden was also expressed as workload inequity. Participants described consistent patterns in which junior clinicians and risk managers carried the bulk of investigative labour, while senior clinicians, despite holding critical institutional knowledge, contributed inconsistently (Liepelt *et al*., 2023; Yanchus *et al*., 2017). Leaders perceived this imbalance as undermining both the rigour and legitimacy of investigations and reinforcing hierarchical dynamics that inhibit collaborative learning cultures (Iedema *et al*., 2006; Wu *et al*., 2008). Consistent with earlier research (Macrae and Vincent, 2014) and more recent evidence (Crompton *et al*., 2025; McHugh *et al*., 2024), participants advocated for dedicated investigation teams with protected time, specialised training and expertise in systems thinking.

A further, pervasive concern was whether the substantial organisational effort invested in SAE investigations reliably translates into meaningful learning or sustained improvement (Martin-Delgado *et al*., 2020; Panagioti *et al*., 2019). Leaders expressed frustration at the repetition of similar recommendations across recurring incident types, echoing critiques that characterise organisational learning as superficial or cyclical (Bowditch *et al*., 2025; Lea *et al*., 2023). Participants’ accounts suggest that these patterns contribute to organisational amnesia, whereby important lessons are lost or diluted over time due to workforce turnover, shifting priorities and fragmented knowledge sharing systems (Ratnapalan and Uleryk, 2014; Treleaven and Sykes, 2005). Persistent weaknesses were extended to the monitoring of recommendations, evaluation of impact and dissemination of learning across services (Braithwaite *et al*., 2006; Hibbert *et al*., 2018; Kumar *et al*., 2020; Woodier *et al*., 2024), indicating that organisational attention remains disproportionately focused on the administrative priority of timely completion of investigations rather than embedding learning into routine clinical and operational practice with goal of improving care.

Leaders also highlighted the limited capacity of current investigation methodologies, particularly RCA, to account for broader contextual conditions such as staffing shortages, escalating clinical complexity and rapid patient turnover. These critiques align with growing evidence that many investigations fail to adequately incorporate systems thinking, instead privileging procedural compliance over structural insight (Goekcimen *et al*., 2023; Machen, 2023). Participants strongly supported thematic and multi-incident review approaches, which allow organisations to identify cross-cutting vulnerabilities and systemic patterns rather than treating events as isolated anomalies (Anderson and Watt, 2020). Internationally, such aggregated learning is increasingly recognised as essential for improving safety and resilience at scale.

Legislated protection emerged as a focal point of tension and debate within the investigation process. While some leaders viewed legislative protection as essential for promoting openness and candour within investigation interviews, psychological safety and honest staff participation (Studdert *et al*., 2010), others argued that it constrained transparency, limited open disclosure and risked eroding public trust (Fukami, 2024; Gille *et al*., 2021; McHugh *et al*., 2024). Restrictions on report content and the inability to share contextual learning were perceived as significant barriers to organisational improvement (Bligh *et al*., 2018; Park *et al*., 2023; Wood *et al*., 2024; Woodward, 2006). These divergent views underscore the need for more nuanced legislative frameworks that balance the benefits of protected candour with the imperative for transparency and system-level learning (Kachalia, 2013).

Despite the inequitable distribution of investigative labour, leaders also identified the involvement of highly paid professional groups, namely psychiatrists, medical consultants and senior executives, as a substantial contributor to the overall cost of SAE investigations (Pereira *et al*., 2025). While such involvement provides essential clinical oversight, governance authority and risk management expertise, it simultaneously amplifies the financial and administrative burden associated with increasingly complex investigation and assurance processes (Taitz *et al*., 2010).

Within these themes, the findings highlight the significant challenges leaders face in operationalising Just Culture and Restorative Practice (Boskeljon-Horst *et al*., 2024; Dekker *et al*., 2011; Dekker and Breakey, 2016) within SAE investigation systems. Although leaders expressed strong conceptual commitment to fairness, psychological safety, transparency and learning inherent within these principles, they described substantial barriers to embedding these principles in everyday practice (Kuipers *et al*., 2021). Competing organisational priorities, medico-legal risk, entrenched hierarchies and resource constraints frequently undermined aspirations for openness and relational repair (Kumah *et al*., 2025; Peerally *et al*., 2017). Restorative approaches, in particular, were viewed as requiring time, skilled facilitation, conditions rarely available in overstretched systems (Boskeljon-Horst *et al*., 2024; Dekker *et al*., 2011; Dekker and Breakey, 2016).

## Conclusion

SAE investigations methods were described as occupying a complex governance space, providing organisational assurance and accountability while generating substantial resource, financial and workforce burdens. Leaders expressed ongoing uncertainty about the extent to which current investigation approaches deliver sustained safety improvement, noting recurring recommendations, limited dissemination of learning and variable investigative capability. Legislated protection was experienced as enabling candour while simultaneously constraining transparency. Together, these insights point to the need for investigation systems that are proportionate, fit for industry needs, and more closely aligned with meaningful organisational learning and sustainable safety improvement.

## Study limitations

Several limitations should be considered. First, the study was limited to a small sample of senior leaders, excluding private health services. While the sample size was sufficient for thematic saturation, it may limit transferability of findings to other contexts. Second, broad recruitment was constrained by ethics and site-specific approval requirements and restriction of participation by leaders in eastern Australian states, potentially limiting the range of perspectives included. Third, interviews were conducted online and by a single researcher, which may have limited opportunities to capture non-verbal cues and introduced potential interpretive bias despite efforts to ensure rigour. Lastly, interviews were restricted to 30 min potentially limiting expansion and depth of conversation.
